# *Clostridium butyricum* WL-53 Ameliorates High-Fat-Diet-Induced Obesity and Inflammatory Response in Mice by Regulating the Intestinal Microbiota and Liver Metabolism

**DOI:** 10.3390/foods15091599

**Published:** 2026-05-05

**Authors:** Qiuyan Li, Qianqian Wang, Yaqin Tang, Peiyun Gao, Cunxi Nie, Junli Niu, Wenju Zhang

**Affiliations:** College of Animal Science and Technology, Shihezi University, North Street 4, Shihezi 832000, China; qiuyanli2025@outlook.com (Q.L.); 15864653585@163.com (Q.W.); t892585470@sina.com (Y.T.); 13654656106@163.com (P.G.); niecunxi@shzu.edu.cn (C.N.)

**Keywords:** *Clostridium butyricum*, obesity, gut microbiota, inflammation

## Abstract

This study induced obesity in mice through a high-fat diet (HFD) to investigate the regulatory effects of *Clostridium butyricum* WL-53 (*C. butyricum* WL-53) on lipid metabolism and intestinal inflammation. Thirty 6-week-old male C57 mice were randomly divided into three groups: the normal diet group (ND), the high-fat diet group (HFD), and the HFD supplemented with *Clostridium butyricum* (CB, *C. butyricum*) group (HFD-CB). The experiment lasted for five weeks. The results demonstrated that mice in the HFD-CB group exhibited significantly alleviated weight gain, reduced fat mass, and decreased hepatic lipid deposition. *C. butyricum* WL-53 treatment improved serum and hepatic lipid markers (TC, TG), decreased the levels of pro-inflammatory factors (TNF-α, IL-1β), and increased those of anti-inflammatory factors (IL-10, IL-4). Gut microbiota analysis indicated that HFD reduced microbial diversity and increased the abundance of *Firmicutes*. Meanwhile, *C. butyricum* WL-53 intervention reversed these changes and enriched beneficial genera. Metabolomics analysis revealed that *C. butyricum* WL-53 regulated glycerophospholipid metabolism, arachidonic acid metabolism, and cAMP signaling pathways, reversing metabolites to ameliorate lipid deposition and inflammation. In summary, *C. butyricum* WL-53 alleviates HFD-induced obesity and inflammation via gut microbiota modulation and metabolic reprogramming.

## 1. Introduction

Over the past decade, the global prevalence of overweight has risen substantially, establishing obesity as a significant public health challenge in numerous countries [1,2]. This trend is observed not only among adults but also among adolescents and children [3]. It parallels the global increase in the incidence, mortality, and disability associated with metabolism-related diseases, including type 2 diabetes, hypertension, and non-alcoholic fatty liver disease [4]. Furthermore, obesity, as a chronic condition, is closely linked to systemic low-grade inflammation, which may precipitate various metabolic disorders over time. Consequently, the prevention and management of obesity represent critical priorities in public health.

In recent years, the gut microbiota has emerged as a promising target for obesity treatments. Dietary and environmental conditions can contribute to increased adiposity or overweight status, which subsequently alters the gut microbiota, resulting in metabolic abnormalities and inflammation [5]. The gut, liver, and immune system maintain a symbiotic relationship with the gut microbiota. Disruption of this microbial balance by a high-fat diet (HFD) impairs intestinal barrier function and contributes to liver disease via the gut–liver axis [6]. Obesity and intestinal inflammation exhibit a complex bidirectional regulatory relationship, forming a vicious cycle driven by impaired intestinal barrier function, dysbiosis, immune activation, and metabolic disorders [7]. This interplay within the “gut-body axis” mutually exacerbates both conditions, collectively facilitating the progression of obesity and associated disorders such as insulin resistance and hepatic steatosis.

Currently, probiotics have become the most widely recognized approach for improving gut microbiota and treating dysbiosis. They may work through pathways such as the “gut–brain axis”, promoting beneficial bacterial proliferation, inhibiting harmful bacterial overgrowth [8], strengthening the epithelial barrier, suppressing pathogens, and facilitating short-chain fatty acid (SCFA) production, thereby restoring a “lean gut microbiota”. Additionally, probiotics help reduce fat deposition and improve lipid profiles [9], and thereby support weight management and obesity prevention. Furthermore, probiotics not only have positive effects in intervening to reduce cognitive impairments associated with obesity and HFD, but they can also alleviate obesity-related metabolic diseases and inflammation through mechanisms such as increasing anti-inflammatory adipokines in the blood, reducing pro-inflammatory adipokines to regulate glucose and fatty acid breakdown, improving insulin sensitivity, and reducing systemic inflammation [10,11]. Consistent with these findings, research has shown that probiotics improve the health of overweight and obese postmenopausal women [12] and reduce intestinal inflammation in obese adults and alleviate childhood obesity [13]. Notably, Guo et al. reported that *Lactiplantibacillus plantarum* BGI-N6 remodels the gut microbiota and improves obesity-induced inflammation and oxidative stress through gut microbiota-mediated metabolites [14]. Collectively, probiotics hold considerable therapeutic potential for obesity-related interventions.

*Clostridium butyricum* (CB, *C. butyricum*) is a Gram-positive, anaerobic, spore-forming bacterium known for its probiotic properties. It produces SCFAs, such as butyrate, lactate, and acetate, as well as various antimicrobial compounds. Supplementing with *C. butyricum* can enhance immune function, promote gut microbiota and intestinal health [15,16], and prevent colonic barrier damage and inflammation [17]. Although previous research has indicated that oral administration of *C. butyricum* can mitigate obesity induced by HFD [18], the specific therapeutic potential of the WL-53 strain, along with the mechanisms through which it modulates lipid metabolism and inflammation related to obesity, remains insufficiently elucidated.

To create an obese mouse model, this study feeds animals a high-fat diet and uses the WL-53 strain of *C. butyricum* (*C. butyricum* WL-53) to investigate its effects on fat metabolism, HFD-induced early inflammation, and gut microbiota in obese mice. The aim is to provide a reference for subsequent use of *C. butyricum* to ameliorate obesity, obesity-induced intestinal inflammation, and other metabolic diseases.

## 2. Materials and Methods

### 2.1. Experimental Animals and Diet

All male C57 mice at six weeks of age were maintained in the animal facility of Shihezi University’s College of Animal Science and Technology.

Two diets were employed: a high-fat diet (60% kcal from fat) and a standard chow diet (10% kcal from fat). Both the mice and the two diets were purchased from Xinjiang Hengchao Biotechnology Co., Ltd (Shihezi, China). The full ingredient profiles and nutritional specifications of both diets are detailed in Table 1.

*C. butyricum* was obtained from Qingdao Weilan Biological Co., Ltd. (Qingdao, China) with the strain number WL-53.

### 2.2. Experimental Design

After adapting to a standard diet for 1 week, 30 mice were randomly assigned to three groups, with 10 animals per group. The blank control group (standard diet group, ND) continued to receive the standard diet; the experimental control group (high-fat diet group, HFD) was given a high-fat diet; and the treatment group (HFD-CB) received the high-fat diet and additionally gavaged daily with oral gavage of 1 × 10^9^ CFU of *Clostridium butyricum* WL-53.

Daily oral administration of 0.2 mL sterile phosphate-buffered saline (PBS) was performed on both the ND and HFD group animals. In contrast, the HFD-CB group was given 0.2 mL of a freshly made sterile suspension of *C. butyricum* WL-53 in PBS. The bacterial suspension was prepared daily by suspending *C. butyricum* powder in sterile PBS. The viable count of *C. butyricum* WL-53 was determined via the standard plate count method on reinforced clostridial medium (RCM) agar, and the final concentration was adjusted to 1 × 10^9^ CFU/mL. The experiment lasted for 5 weeks. All treatments were administered daily at 10:00 AM, and the animals were provided with unrestricted access to food and water for the duration of the experiment. Experimental animal use in this study received approval from Shihezi University’s Animal Welfare Committee (Shihezi, China; Ethics Approval No: 2025-909).

### 2.3. Sample Collection and Indicator Measurement

All animal procedures, including body weight assessments, sample collection, and biochemical analyses, were conducted under blinded conditions to reduce observer bias.

#### 2.3.1. Body Weight Gain and Visceral Fat Weight

Throughout the trial, the general growth conditions of the mice were monitored daily, with food intake recorded every day, and their weights were measured weekly to calculate weight gain. Upon completion of the experiment, five mice were randomly selected from each group and euthanized by cervical dislocation. After dissection, their visceral adipose tissue was harvested, rinsed with sterile saline, blotted dry, weighed, and recorded.

#### 2.3.2. Blood Parameter Testing

Five randomly selected mice from each group had their orbital venous plexuses sampled once at the conclusion of the experiment and prior to euthanasia. Samples were placed at ambient temperature for 1 h to equilibrate, after which they were centrifuged at 3000 rpm and 4 °C for 10 min. The obtained serum was then frozen at −20 °C for later use.

Serum concentrations of blood glucose (BG), triglycerides (TG), total cholesterol (TC), low-density lipoprotein (LDL), and high-density lipoprotein (HDL) were quantified using a commercial assay kit. Additionally, serum inflammatory cytokines, including interleukin-1β (IL-1β), interleukin-6 (IL-6), and tumor necrosis factor-α (TNF-α), were measured using an enzyme-linked immunosorbent assay (ELISA) kit (Shanghai Yuanju Biological Technology Co., Ltd., Shanghai, China).

#### 2.3.3. Measurement of Visceral Fat Indicators

After weighing, visceral fat tissue samples of all groups were promptly fixed in 4% paraformaldehyde. They were then washed with distilled water, dehydrated through an ethanol gradient, and cleared by sequential immersion in xylene for 10 min and 7 min. After paraffin embedding, 5-μm-thick sections were cut. The resulting slides underwent hematoxylin and eosin (H&E) staining and were examined under a light microscope.

#### 2.3.4. Colonic Cytokine and Microbiota Analysis

Using a commercial ELISA kit (Shanghai Yuanju Biological Technology Co., Ltd., Shanghai, China), colonic tissues from each group were measured for TNF-α, interleukin-10 (IL-10), and interleukin-22 (IL-22). A portion of the colonic contents was collected in cryotubes, flash-frozen in liquid nitrogen promptly, and reserved at −80 °C for future analyses.

The E.Z.N.A.^®^ Soil DNA Kit (Omega Bio-tek, Norcross, GA, USA) served to isolate microbial DNA from mouse colonic contents. DNA integrity and yield were checked by 1.0% agarose gel electrophoresis, and concentrations were measured with a NanoDrop 2000 (Thermo Scientific, Waltham, MA, USA). Instrument settings: 220–320 nm scan, 10 mm path, 25 °C. Its built-in software automatically computed A260/A280 and A260/A230 ratios to assess purity and yield. Qualified DNA was kept at −80 °C. The 16S rRNA gene’s V3–V4 region was amplified with primers 338F (5′-ACTCCTACGGGAGGCAGCAG-3′)/806R (5′-GGACTACHVGGGTWTCTAAT-3′) on a T100 Thermal Cycler (Bio-Rad, Hercules, CA, USA). Purified amplicons were equimolarly pooled and sequenced on an Illumina NextSeq 2000 platform (Illumina, San Diego, CA, USA) following Majorbio’s (Shanghai, China) standard protocols. High-quality reads were clustered into operational taxonomic units (OTUs) at 97% similarity via UPARSE v7.1. Taxonomic assignment aligned representative sequences to a bacterial database; community profiles were generated at multiple levels. OTU proportions and alpha diversity indices were calculated to infer species richness and evenness. Linear discriminant analysis effect size (LEfSe) (Majorbio Cloud Platform, https://cloud.majorbio.com, accessed on 23 May 2025) was used to compare microbial communities across groups and identify biomarkers. Functional potential was predicted by PICRUSt and annotated with Gene Ontology (GO). Raw sequences are available in NCBI SRA under accession PRJNA1304187.

#### 2.3.5. Liver Tissue Analysis

Mice in each group had their liver tissue samples taken. A portion of each liver was immediately placed in 4% paraformaldehyde and then subjected to the same histological processing as described for visceral fat samples (dehydration, clearing, embedding, sectioning, H&E staining, and light microscopy). Separately, placed liver tissue samples were aliquoted into cryotubes and stored at −20 °C for subsequent quantification of TG, TC, and free fatty acid (FFA) levels, performed according to the manufacturers’ instructions of the respective assay kits. Another portion of liver tissue was instantly frozen in liquid nitrogen, maintained at −80 °C, and later subjected to non-targeted metabolomics analysis.

For metabolite extraction, 100 mg of frozen liver sample was combined with 800 μL of methanol/water (4:1, *v*/*v*) spiked with four internal standards (including 0.02 mg/mL L-2-chlorophenylalanine). The samples were homogenized (50 Hz, 6 min, −10 °C), sonicated (40 kHz, 30 min, 5 °C), and centrifuged (13,000× *g*, 15 min, 4 °C), and the supernatant was collected for analysis. Chromatographic separation was performed on an ultra-high-performance liquid chromatography (UHPLC)-Orbitrap Exploris 240 system equipped with an ACQUITY HSS T3 column, using a 12-minute gradient elution program optimized for both ionization modes: mobile phase B was maintained at 0% from 0 to 0.2 min, increased to 25% from 0.2 to 3 min, ramped to 100% from 3 to 9 min, held at 100% B for 1 min (over 9 to 10 min), rapidly returned to 0% over 10 to 10.1 min, and kept at 0% B until the end of the run at 12 min. Mass spectrometric detection was carried out using an ESI source in positive/negative modes, with the following key parameters: source temperature 400 °C, sheath gas 40 AU, auxiliary gas 10 AU, ISVF 2800 V (negative) and 3500 V (positive). MS/MS fragmentation employed a normalized collision energy of 4 and stepped energies of 20, 40, 60 V, and data were acquired in data-dependent acquisition (DDA) mode over *m*/*z* 70–1050. All other experimental details (e.g., grinding instrument model and column specifications) were as described in a previous study [19].

#### 2.3.6. Analysis of the Correlation Between Microorganisms and Metabolites

The Majorbio Cloud Platform was employed to analyze correlations between LEfSe differential microorganisms and differential metabolites. Initially, a two-way orthogonal partial least squares (O2PLS) model was constructed to assess overall consistency analysis. Then, a correlation analysis was conducted between metabolites and microorganisms at the overall level. Metabolite data were dimension-reduced and clustered using Hierarchical clustering (HCLUST); finally, metabolites and microorganisms within the same group were quantified by calculating Pearson correlation coefficients.

### 2.4. Data Statistics and Analysis

For growth performance data, all 10 mice in each group were measured individually. For cytokine detection, lipid metabolism indicators, 16S rRNA gene sequencing, and liver metabolomics, five mice per group were randomly selected, and all assays were performed in three technical replicates to ensure data precision and repeatability. SPSS 26.0 and GraphPad Prism 8.0.2 were used for all statistical evaluations. One-way ANOVA was employed to test for significance, and multiple comparisons were subsequently performed using the LSD approach. All data are presented as the mean ± standard error of the mean (SEM). Differences with *p* < 0.05 were regarded as statistically significant.

## 3. Analysis of Results

### 3.1. Effects of Clostridium butyricum WL-53 on Body Weight and Visceral Fat Weight in Mice

**Figure 1:**
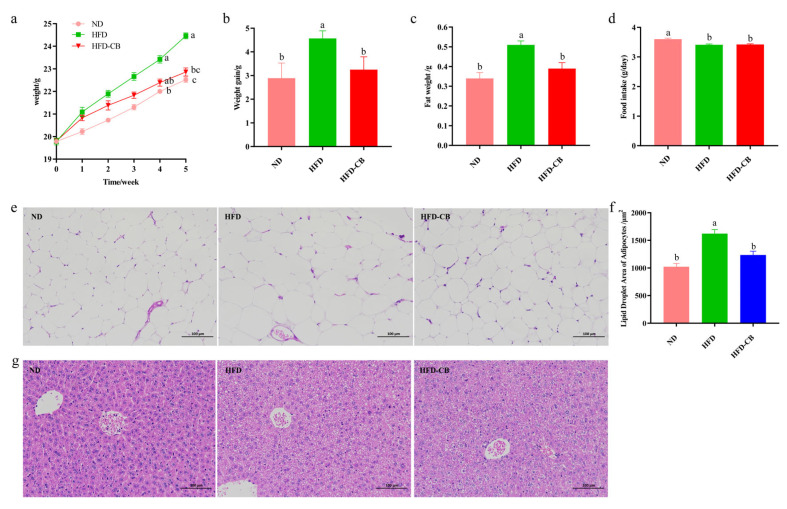
Effects of *Clostridium butyricum* WL-53 on growth parameters in mice. (**a**) Body weight changes (*n* = 10); (**b**) Body weight gain (*n* = 10); (**c**) Fat weight (*n* = 10); (**d**) Daily food intake (*n* = 10); (**e**) Fat cell sections (scale bar = 100 μm) (*n* = 5); (**f**) Size of liver fat cells (*n* = 10); (**g**) Liver tissue sections (scale bar = 100 μm) (*n* = 5). Significant differences are marked by distinct letters (*p* < 0.05), whereas identical letters denote no statistical difference. Abbreviations: ND, standard diet group; HFD, high-fat diet group; HFD-CB, high-fat diet treated with *Clostridium butyricum* WL-53 group.

Body weights increased over time in all treatment groups (Figure 1a). Mice receiving the HFD showed significantly greater weight gain and visceral fat mass than those in the ND group (*p* < 0.05) (Figure 1b,c). In contrast, no statistically significant differences in these two parameters were detected between the HFD-CB and ND groups (*p* > 0.05) (Figure 1b,c). Among the groups fed a high-fat diet, mean daily food intake remained comparable (*p* > 0.05); however, all HFD-fed groups consumed markedly less food per day than the ND group (*p* < 0.05) (Figure 1d).

Compared with the ND and HFD-CB groups, the HFD group displayed a greater adipocyte size (Figure 1e). The adipocyte area in the HFD-CB group did not differ significantly from that in the ND group and was significantly smaller than that in the HFD group (Figure 1f). Additionally, mice in the ND and HFD-CB groups had fewer liver fat droplets, whereas the HFD group exhibited prominent white lipid droplets (Figure 1g).

### 3.2. Effects of Clostridium butyricum WL-53 on Serum and Liver Lipid Metabolism-Related Indicators in Mice

Serum concentrations of BG, TC, TG, and LDL were significantly elevated in the HFD group than those in the ND and HFD-CB groups (*p* < 0.05), and there was no significant difference between the latter two groups (*p* > 0.05). Across all treatment groups, HDL levels remained comparable (*p* > 0.05) (Table 2).

Regarding liver levels, the HFD group exhibited significantly elevated TC, TG, and FFA compared with the ND and HFD-CB groups (*p* < 0.05). Meanwhile, there was no statistically significant difference between the ND and HFD-CB groups (*p* > 0.05) (Table 2).

### 3.3. Effects of Clostridium butyricum WL-53 on Serum and Colonic Cytokine Levels in Mice

In comparison with the ND group, the HFD group had significantly increased serum levels of TNF-α and IL-1β (*p* < 0.05), whereas IL-4 concentrations were markedly reduced (*p* < 0.05). No notable changes in IL-10 content were detected between the two groups (*p* > 0.05). By contrast, when compared with the HFD group, the HFD-CB group showed markedly reduced TNF-α and IL-1β levels (*p* < 0.05) along with elevated IL-10 and IL-4 concentrations (*p* < 0.05). Meanwhile, no statistically significant differences were observed between the HFD-CB and ND groups (*p* > 0.05) (Table 3).

In the colon, TNF-α levels were significantly higher in the HFD group than in the ND group (*p* < 0.05), while IL-10 and IL-22 concentrations were markedly reduced (*p* < 0.05). Compared with the HFD group, the HFD-CB group exhibited notably lower TNF-α levels (*p* < 0.05) and higher IL-22 levels (*p* < 0.05). A tendency toward higher IL-10 levels was observed in the HFD-CB group, although this difference did not achieve statistical significance (*p* > 0.05). For all colonic cytokines measured, no statistically significant differences were found between the HFD-CB and ND groups (*p* > 0.05) (Table 3).

### 3.4. Analysis of Colonic Microbial Diversity in Mice

**Figure 2:**
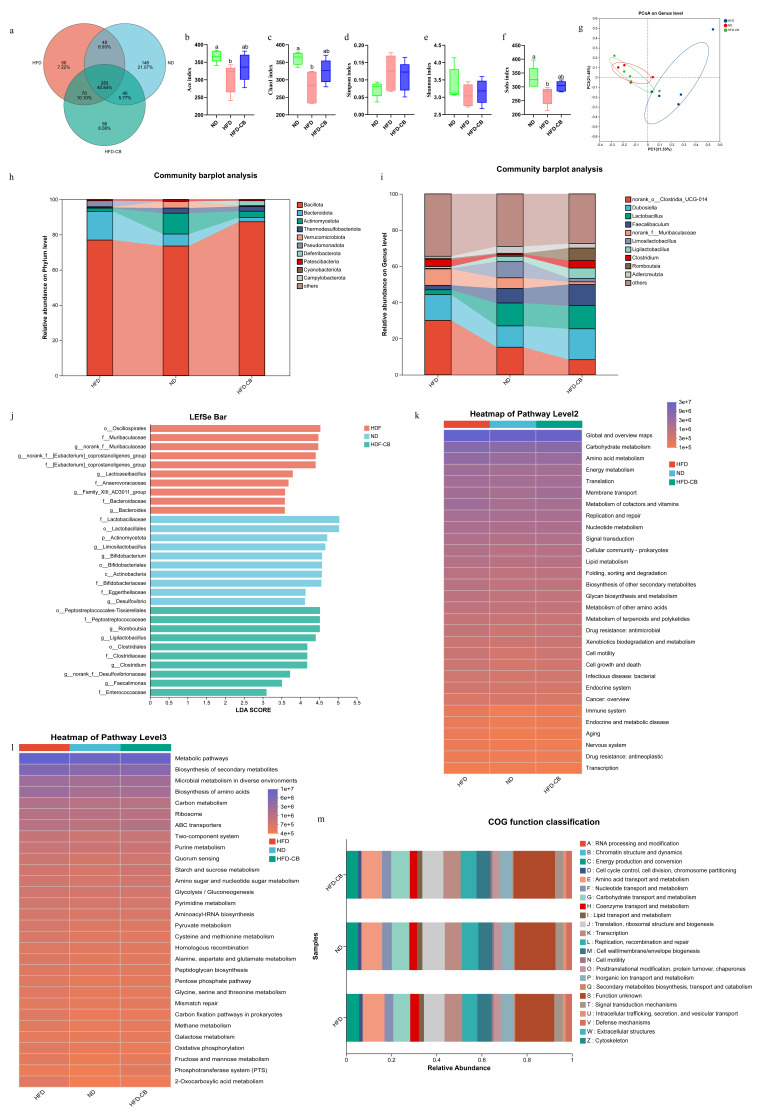
Impact of *Clostridium butyricum* WL-53 on the gut microbiota of mice (*n* = 5). (**a**) Species composition Venn diagram; (**b**) ACE; (**c**) Chao1; (**d**) Simpson; (**e**) Shannon; (**f**) Sobs; (**g**) PCoA analysis plot; (**h**) Effects on relative abundance of microorganisms at the phylum level; (**i**) Effects on relative abundance of microorganisms at the genus level; (**j**) LEfSe analysis LDA scores of microbial communities; (**k**) KEGG functional prediction at the secondary classification level; (**l**) KEGG functional prediction at the tertiary classification level; (**m**) COG functional prediction. Significant differences are marked by distinct letters (*p* < 0.05), whereas identical letters denote no statistical difference. Abbreviations: ND, standard diet group; HFD, high-fat diet group; HFD-CB, high-fat diet treated with *Clostridium butyricum* WL-53 group; PCoA, principal coordinates analysis; LEfSe, linear discriminant analysis effect size; KEGG, Kyoto encyclopedia of genes and genomes; COG, clusters of orthologous groups.

The mouse samples across the three experimental groups shared 283 OTUs, constituting 40.84% of all operational taxonomic units detected. The ND group exhibited 146 unique OTUs, while the HFD-CB and HFD groups contained 50 and 56 unique OTUs, respectively (Figure 2a). The HFD group showed significantly reduced ACE (Figure 2b), Chao1 (Figure 2c), and Sobs (Figure 2f) indices relative to the ND group (*p* < 0.05). However, the ND and HFD-CB groups did not differ significantly from each other (*p* > 0.05). For the Simpson index (Figure 2d) and the Shannon index (Figure 2e), no significant differences were identified among the experimental groups (*p* > 0.05). As revealed by principal coordinates analysis (PCoA, Figure 2g), the samples from each group formed tight clusters.

The dominant phyla in the HFD group were *Bacillota*, *Bacteroidota,* and *Pseudomonadota*, while the HFD-CB and ND groups were dominated by *Bacillota*, *Actinomycetota*, and *Bacteroidota*. *Actinomycetota* abundance was reduced in the HFD group, whereas it partially recovered in the HFD-CB group following *C. butyricum* WL-53 treatment (Figure 2h). At the genus level, norank_o_*Clostridia_UCG-014*, *Dubosiella*, and norank_f_*Muribaculaceae* dominated in the HFD group, whereas the HFD-CB and ND groups were mainly populated by norank_o_*Clostridia_UCG-014*, *Dubosiella*, *Lactobacillus*, and *Faecalibaculum*. The beneficial genera, including *Lactobacillus*, *Faecalibaculum*, *Limosilactobacillus*, and *Ligilactobacillus*, declined in abundance under HFD feeding but showed a marked recovery in the HFD-CB group (Figure 2i). LEfSe differential analysis revealed that the relative abundance of f_*Lactobacillales*, p_*Actinomycetota*, g_*Limosilaclobacillus*, and g_*Bifidobacterium* were significantly higher in the ND group than in the other two groups. Significantly higher abundances of o_*Oscillospirales*, f_*Muribaculaceae*, and g_norank_f_*[Eubacterium]_coprostanoligenes_group* were observed in the HFD group, while the HFD-CB group showed significantly elevated levels of o_*Peptostreptococcales-Tissierellales*, f_*Peptostreptococcaceae*, g_*Romboutsia*, and g_*Ligilactobacillus* (Figure 2j).

According to Kyoto Encyclopedia of Genes and Genomes (KEGG) functional prediction at level 2 (Figure 2k), the three most enriched microbial pathways were Carbohydrate metabolism, Amino acid metabolism, and Energy metabolism. At pathway level 3 (Figure 2l), the top three enriched pathways in microorganisms were Metabolic pathways, Biosynthesis of secondary metabolites, and Microbial metabolism in diverse environments. The gut microbiota of mice primarily involves 23 Clusters of Orthologous Groups (COG) functions (Figure 2m). The most abundant functions were related to fundamental microbial processes, including Translation, ribosomal structure and biogenesis, Replication, recombination and repair, Cell wall/membrane/envelope biogenesis, Energy production and conversion, and Inorganic ion transport and metabolism.

### 3.5. Liver Non-Targeted Metabolomics Analysis

**Figure 3:**
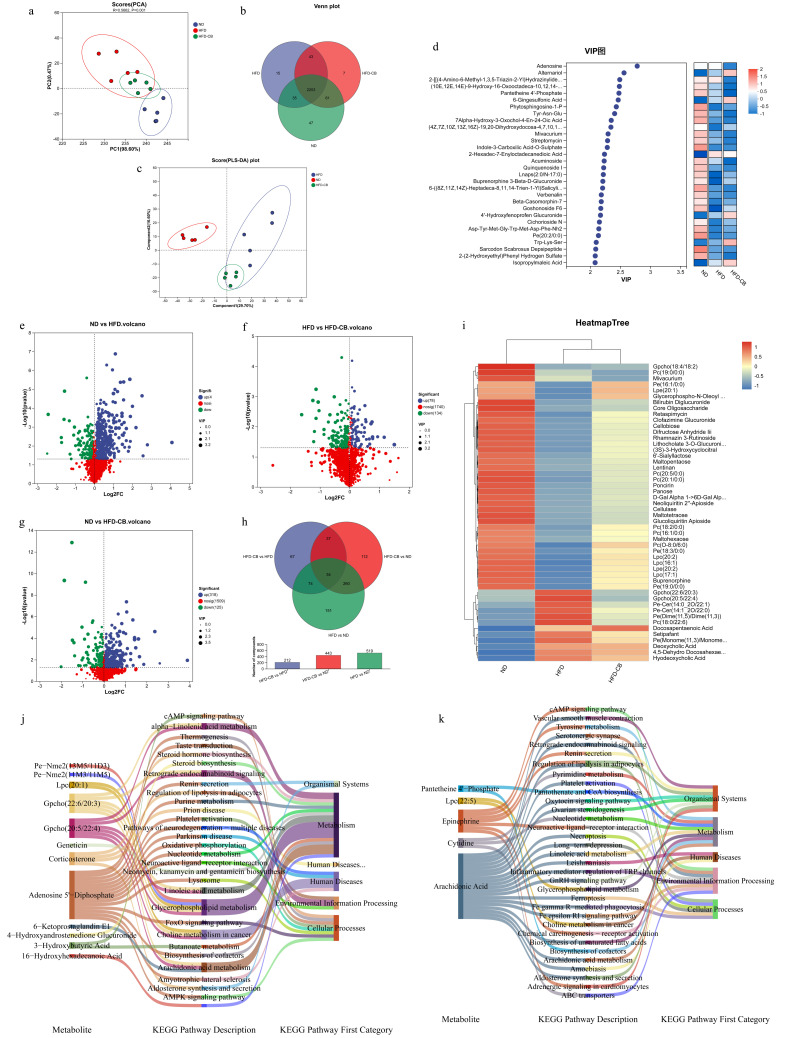
Non-targeted metabolomics analysis of mouse liver tissue (*n* = 5). (**a**) PCA score scatter plot of metabolites; (**b**) Venn diagram of metabolites; (**c**) PLS-DA score scatter plot of metabolites; (**d**) Metabolite VIP analysis bubble plot; (**e**) Volcano plot of differential metabolites between the ND and the HFD group; (**f**) Volcano plot of differential metabolites between the HFD and the HFD-CB group; (**g**) Volcano plot of differential metabolites between the ND and the HFD-CB group; (**h**) Venn diagram of differential metabolites; (**i**) HCLUST heatmap of differential metabolites; (**j**) Sankey diagram of KEGG pathways mapped by differential metabolites between the group-pair of HFD and HFD-CB group, and the group-pair of HFD and ND group; (**k**) Sankey diagram of KEGG pathways mapped by differential metabolites between the group-pair of HFD and HFD-CB groups and the group-pair of HFD-CB and ND group. Abbreviations: ND, standard diet group; HFD, high-fat diet group; HFD-CB, high-fat diet treated with *Clostridium butyricum* WL-53 group; PCA, principal component analysis; PLS-DA, partial least squares discriminant analysis; KEGG, Kyoto encyclopedia of genes and genomes; HCLUST, hierarchical clustering.

PCA analysis showed that mouse samples within each group clustered well (Figure 3a). A total of 2253 metabolites were shared across all groups of mouse liver tissues, with 47 unique to the ND group, 15 unique to the HFD group, and 7 unique in the HFD-CB group (Figure 3b). Based on partial least squares discriminant analysis (PLS-DA), 29.7% of the variance was explained by the first principal component and 16.6% by the second. The distribution of each group was relatively concentrated, and distinct separation between different groups was observed, confirming the reliability and stability of the model (Figure 3c). Based on the VIP values of each metabolite, Adenosine and Alternariol, among others, had higher VIP scores. Numerous metabolites in the HFD group differed significantly from those in the ND group. Expression levels exceeding 1 were observed in the ND group for several metabolites, including Sarcodon Scabrosus Depsipeptide, Pe (20:2/0:0), 6-((8Z, 11Z, 14Z)-Heptadeca-8,11,14-Trien-1-Yl)Salicylic Acid, and Phytosphingosine-1-P, while metabolite expression in the HFD group was generally lower. Metabolites such as Alternariol, 6-Gingesulfonic Acid, Trp-Lys-Ser, and Isopropylmaleic Acid exhibited values close to 1 in the HFD-CB group (Figure 3d).

A total of 1493 metabolites exhibited differential abundance between the ND and the HFD group, with 404 markedly upregulated and 115 down (Figure 3e). Between the HFD and HFD-CB groups, 732 differentially expressed metabolites were identified, comprising 78 and 134 metabolites that were significantly upregulated and downregulated, respectively (Figure 3f). Between the ND and HFD-CB groups, 1352 differentially expressed metabolites were identified, with 318 and 125 differentially expressed metabolites being upregulated and downregulated, respectively (Figure 3g). There were 34 common differentially expressed metabolites among the three groups; 151 such metabolites were identified between the HFD and ND groups, 112 were found between the HFD-CB and ND groups, and 67 between HFD-CB and HFD groups (Figure 3h). The HeatmapTree (Figure 3i) analysis revealed distinct metabolic profiles among the three groups, as visualized by hierarchical clustering. A wide range of metabolites, including carbohydrates and their derivatives, flavonoid glycosides, bile acid-related metabolites, and different glycerophospholipids like Gpcho (18:4/18:2), Pc (19:0/0:0), and Pe (16:1/0:0), were significantly less abundant in the HFD group than in the ND group. In contrast, the HFD group showed marked upregulation of several lipid and bile acid-related metabolites, including Gpcho (22:6/20:3), Pe-Cer (14:0_2O/22:1), Deoxycholic Acid, and Hyodeoxycholic Acid, as well as Setipafant and other related metabolites. Notably, those metabolites downregulated in the HFD group tended to rise in the HFD-CB group, returning stepwise to ND-like levels. Meanwhile, lipid and bile acid metabolites that were elevated under HFD conditions showed a significant reduction in the HFD-CB treatment.

Differential metabolites from comparative groups were aligned to the KEGG pathway database to uncover associated pathways. Between the group pair of HFD and HFD-CB and the group pair of HFD and ND, 12 differential metabolites mapped to 29 pathways (Figure 3j). Most metabolites were primarily enriched in the Metabolism category, including pathways such as Glycerophospholipid metabolism (linked to metabolites like Gpcho(22:6/20:3) and Gpcho(20:5/22:4)), Linoleic acid metabolism, alpha-Linolenic acid metabolism, Arachidonic acid metabolism, Purine metabolism (associated with Adenosine 5′-Diphosphate), and Steroid hormone biosynthesis. Several signaling pathways, including the cAMP and AMPK pathways and the FoxO pathway, were also significantly enriched. Notably, Pe-Nme2 was linked to linolenic acid metabolism and cAMP signaling. Meanwhile, hormones (e.g., Corticosterone, 6-Ketoprostaglandin E1) and lipid metabolites were preferentially enriched in metabolic and cellular process pathways, including arachidonic acid metabolism and AMPK signaling. Between the HFD and HFD-CB group-pair and the HFD-CB and ND group-pair, five differential metabolites mapped to 33 pathways (Figure 3k). The Sankey diagram showed that metabolites, particularly Arachidonic acid and Epinephrine, were mainly enriched in pathways under Metabolism, Environmental Information Processing, and Cellular Processes. Key pathways included Arachidonic acid metabolism, Glycerophospholipid metabolism, and Neuroactive ligand-receptor interaction; Epinephrine is linked to vascular smooth muscle contraction, while Arachidonic acid is associated with inflammation-related pathways.

### 3.6. Correlation Analysis of Colonic Differential Microorganisms and Hepatic Differential Metabolites

**Figure 4:**
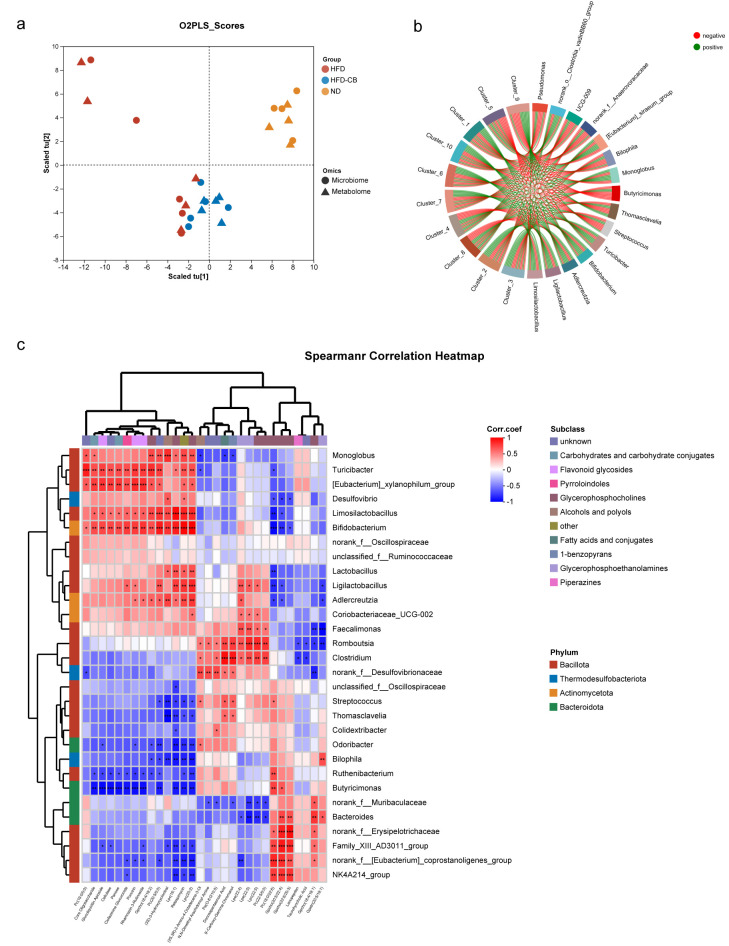
Microbial and metabolite association analysis (*n* = 5). (**a**) O2PLS analysis; (**b**) HCLUST correlation analysis; (**c**) Correlation heat map. Asterisks indicate significant correlations between metabolites and microorganisms: * *p* < 0.05, ** *p* < 0.01, and *** *p* < 0.001. Abbreviations: ND, standard diet group; HFD, high-fat diet group; HFD-CB, high-fat diet treated with *Clostridium butyricum* WL-53 group; O2PLS, orthogonal partial least squares discriminant analysis; HCLUST, hierarchical clustering.

Correlation analysis was performed between LEfSe-identified differential microorganisms (289 taxa) and differential metabolites (407 compounds) at the genus level. The O2PLS model demonstrated a strong association between the datasets, with R^2^ X = 0.5224 and R^2^ Y = 0.4543 (Figure 4a). From the width of the strings in the correlation chord diagram, it was observed that the counts associated with different metabolites or microbes were relatively high. Genera including *Limosilactobacillus*, *Ligilactobacillus*, *Adlercreutzia*, *Bifidobacterium*, and others were primarily negatively correlated with metabolites, whereas *Butyricimonas*, *Thomasclavelia*, *Streptococcus*, *Pseudomonas*, norank_f__*Anaerovoracaceae,* and other genera showed more positive correlations with metabolites (Figure 4b).

Extensive correlations were observed between the top 30 bacterial genera at the genus level at the classification level and the top 30 metabolites by abundance (Figure 4c). Lactic acid bacteria genera including *Limosilactobacillus*, together with *Bifidobacterium* and *Ligilactobacillus*, were significantly positively correlated with Flavonoid glycosides; significant positive correlations were found between Glycerophosphoethanolamines and both *Lactobacillus* and *Coriobacteriaceae_UCG-002*, whereas its relationship with *Desulfovibrio* was significantly negative; multiple genera from the *Bacillota* phylum, including *Romboutsia*, *Faecalimonas*, *Clostridium*, and *Family_XIII_AD3011_group*, were positively correlated with Carbohydrates and carbohydrate conjugates; *Faecalimonas*, *Romboutsia*, and others exhibited significantly positive correlations with Fatty acids and conjugates, while *Faecalimonas*, a genus related to *Peptostreptococcaceae*, showed weakly positive correlations with (3S)-3-Hydroxycyclocitral and (2S,3R)-2-Amino-4-Octadecene-3-Ol in Alcohols and polyols; *Turicibacter* was significantly negatively correlated with Alcohols and polyols.

## 4. Discussion

This study found that feeding a high-fat diet significantly accelerated body weight gain, significantly increased body weight and fat mass, as well as significantly elevated serum and liver levels of blood glucose, total cholesterol, and total triglycerides in mice, consistent with those reported by Wang et al. [20]. This systemic metabolic disturbance, particularly hepatic lipid deposition and hypercholesterolemia, represents the core features of obesity and its associated metabolic diseases [21,22]. For example, maintaining gut microbiota balance, ameliorating intestinal inflammation, and reducing endotoxins allow *C. butyricum* to partially alleviate cognitive impairment in obese mice [17]. When combined with rosuvastatin or soluble dietary fiber, it lowers blood lipids, improves gut microbiota, and alleviates liver injury [23,24]. Notably, Liao et al. [25] reported that the anti-obesity activities of *C. butyricum* may occur independently of increased butyrate concentration, suggesting a more complex mechanism involving broader metabolic regulation.

Inflammation serves as a critical link connecting high-fat diets, intestinal dysfunction, and metabolic disorders. HFD disrupts immune homeostasis, manifested by marked elevations in pro-inflammatory mediators TNF-α and IL-1β, and decreases in anti-inflammatory and regulatory cytokines IL-4, IL-10, and IL-22. This imbalance in inflammatory mediators, representing suppressed Th2 immune responses and impaired anti-inflammatory signaling [26], directly compromises intestinal barrier integrity. This facilitates endotoxin translocation, further exacerbating a vicious cycle of systemic inflammation, obesity, and insulin resistance [27,28]. Moreover, gut-derived signals regulate inflammation by controlling T cell accumulation in adipose tissue, thereby exacerbating obesity-related metabolic impairment [29,30]. *C. butyricum* and other probiotics alleviate inflammation by reducing pro-inflammatory cytokines, including TNF-α, IFN-γ, and IL-1β, while increasing immunoregulatory factors such as IL-10 [28,31,32]. These results are consistent with the present study, in which inflammatory factor levels in the HFD-CB group returned to normal ranges.

The gut microbiota plays a causal role in obesity and related metabolic disorders [33]. The HFD group exhibited reduced microbial diversity, structural imbalance, and decreased levels of beneficial bacteria, including *Actinomycetota*, *Lactobacillus*, and *Faecalibaculum*. This imbalanced gut ecosystem not only affects nutrient acquisition and energy metabolism but also represents a potential root cause of lipid metabolism disorders [34]. Supplementation with *C. butyricum* WL-53 effectively ameliorated this HFD-induced dysbiosis. It largely restored the abundance of beneficial bacteria, including *Lactobacillus* and *Ligilactobacillus*, in the HFD-CB group, while also enriching beneficial bacteria, such as *Faecalibaculum* and *Romboutsia*. Previous studies have indicated that *Romboutsia* may participate in energy metabolism by degrading carbohydrates [35]. Meanwhile, *Faecalibaculum* can enhance intestinal barrier function to reduce endotoxin translocation [36], alleviate systemic inflammation, and attenuate hepatic lipid peroxidation [37], thereby alleviating HFD-induced adverse effects. Furthermore, significant differences were observed in microbes such as *Peptostreptococcales-Tissierellales* and *Peptostreptococcaceae* in the HFD-CB group. Indoleacrylic acid, produced by *Peptostreptococcaceae*, and butyrate, generated by *C. butyricum*, can synergistically enhance intestinal barrier function. Meanwhile, Liu et al. [38] also found that *C. butyricum* combined with inulin significantly enriched *Peptostreptococcaceae* and improved lipid metabolism, indicating a potential synergistic interaction between *C. butyricum* WL-53 and *Peptostreptococcaceae*. Future investigations employing fecal microbiota transplantation to investigate the role of *C. butyricum*-modulated gut microbial communities in obesity and identify functional factors of *C. butyricum* will provide more useful insights into microbial-host interactions in obesity.

HFD induced extensive hepatic metabolic disorders, and elevated levels of Deoxycholic Acid and Hyodeoxycholic Acid reflected abnormalities in the gut–liver axis or disturbances in hepatic bile acid synthesis and metabolism, which are closely related to the influence of the gut microbial community and its metabolites on host metabolism [39,40]. *C. butyricum* WL-53 intervention reversed these disorders by affecting pathways including Glycerophospholipid metabolism, Arachidonic acid metabolism, Linoleic acid metabolism, and ABC transporters. As a precursor of inflammatory mediators, arachidonic acid is closely associated with inflammatory responses when its metabolism is dysregulated [41], and the cAMP signaling pathway is a key regulator of various physiological processes, including glucolipid metabolism, inflammation, and cell growth [42]. Inflammation is a key driver of metabolic diseases induced by HFD, such as insulin resistance and non-alcoholic fatty liver disease [41,43]. In the present study, metabolites including corticosterone and 6-ketoprostaglandin E1 were enriched in the AMPK and cAMP signaling pathways, which are central hubs for energy metabolism and inflammation. Meanwhile, the levels of Adenosine and certain peptides, like Tyr-Asn-Glu, were elevated in the HFD-CB group. These metabolites are known to stimulate intestinal epithelial cell turnover and regeneration, thereby enhancing gut barrier function [44]. These core metabolite changes were associated with pathways categorized under “Enrichment in Environmental Information Processing” and “Steroid hormone biosynthesis”. *C. butyricum* intervention was associated with improved adaptation to HFD [45] and regulated steroid hormone synthesis to influence obesity-related endocrine systems, thereby modulating the body’s gut microbiota [46], energy metabolism, and inflammatory responses, which is consistent with the findings of the present study.

**Figure 5:**
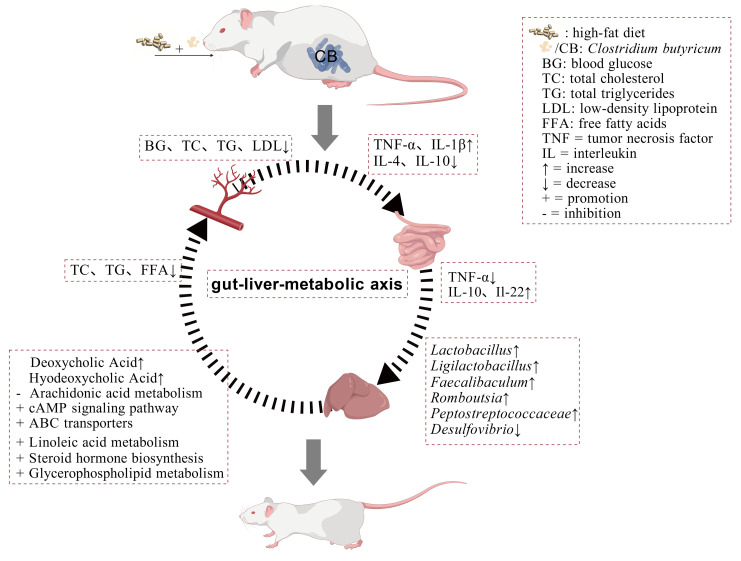
*Clostridium butyricum* WL-53 alleviates the effects of HFD on mice by regulating the gut–liver axis.

The gut–liver axis serves as a critical pathway linking gut microbiota to hepatic metabolic disorders. HFD shifts the microbial metabolic patterns from a carbohydrate-dominant state to a cofactor/vitamin-dependent state, leading to disordered host energy acquisition and exacerbating metabolic inflammation. These observations are consistent with previous findings that HFD impairs microbial carbohydrate metabolism and remodels interactions between bile acids and SCFAs [47,48]. In the present study, *Romboutsia* and *Faecalimonas* exhibited positive correlations with carbohydrates and carbohydrate conjugates. *C. butyricum* enriched bacteria, such as *Romboutsia,* that efficiently degrade complex carbohydrates. Their metabolites may serve as precursors and, together with co-enriched *Faecalimonas*, positively regulate host bile acid synthesis pathways [35,49], thereby inhibiting hepatic lipid absorption via the gut–liver axis. Meanwhile, short-chain fatty acids, such as butyrate produced by carbohydrate metabolism, can activate the GPR43 receptor to directly repair the intestinal barrier [50]. Yan et al. [51] demonstrated that *C. butyricum* exerts hepatoprotective effects in type 2 diabetes mellitus (T2DM) as an effective nutritional intervention via the gut–liver axis, findings consistent with those of the present study. Glycerophosphoethanolamines (GPEA) were positively correlated with *Lactobacillus* and negatively correlated with *Desulfovibrio*. *Lactobacillus* can promote phospholipid synthesis by activating the Akt/mTOR signaling pathway [52]. Concurrently, *C. butyricum* WL-53 reduces the abundance of *Desulfovibrio*, thereby decreasing the inhibitory effect of its metabolite, hydrogen sulfide (H_2_S), on phospholipid synthesis [53], thereby helping correct GPEA metabolic disorder. Furthermore, *Limosilactobacillus* may exert its anti-inflammatory effects by degrading flavonoid glycosides to produce aglycones, thereby inhibiting the NF-κB pathway *o* [54,55,56]. Collectively, *C. butyricum* WL-53 exerts its beneficial effects mainly by targeting the regulation of gut microbiota, reshaping the interaction network between gut microbiota and metabolites, and ultimately modulating systemic lipid metabolism and inflammatory states through the gut–liver metabolism axis (Figure 5).

## 5. Limitations of the Study

First, the study used the C57 mouse model and the WL-53 strain. Due to individual variability and strain specificity, the findings may not fully reflect differences across species, strains, or individuals, nor can they fully represent the effects of other bacterial strains. Furthermore, the experiment included only 6-week-old male mice; therefore, the applicability of the results to different sexes and age groups may be limited. Consequently, the applicability of these results to different sexes and age groups may be limited. Moreover, the 5-week intervention period primarily reflects early-stage effects and does not assess the sustained efficacy of *C. butyricum* on long-term obesity and related chronic inflammation. Finally, although this study uncovered an overall association between the gut microbiota and the host, it did not identify or mechanistically validate specific functional factors (e.g., proteins or metabolites) through which *C. butyricum* may exert its effects. Those will be an important direction for future research.

## 6. Conclusions

The present work confirmed that *C. butyricum* WL-53 treatment significantly mitigates high-fat diet-induced obesity and inflammation responses in mice, with marked improvements in body weight and adipose tissue accumulation. It alleviates both intestinal and systemic inflammation by reducing pro-inflammatory factors (TNF-α, IL-1β) and increasing anti-inflammatory cytokines (IL-10, IL-4, IL-22). Simultaneously, it reverses the reduction in gut microbiota diversity and structural imbalance, enriches beneficial genera such as *Lactobacillus* and *Romboutsia*, and restores gut microbial balance. Regulate Glycerophospholipid metabolism, Arachidonic acid metabolism, cAMP signaling pathways, and increase Adenosine levels to reshape hepatic metabolic pathways and improve hepatic metabolic disorders. In summary, by targeting the gut microbiota and regulating the “gut–liver-metabolism axis”, *C. butyricum* WL-53 effectively mitigates obesity and related metabolic abnormalities induced by a high-fat diet. This provides a theoretical basis for its potential application in preventing and treating obesity and associated inflammatory or metabolic diseases.

## Figures and Tables

**Table 1:** Composition and Nutritional Values of Experimental Diets.

Items	Standard Feed	High-Fat Feed
Ingredient Composition/(g/kg)		
Protein (casein, cysteine)	169.74	262.33
Starch, maltodextrin, sucrose	674.5	250.44
Cellulose	38.6	64.61
Fat (soybean oil, lard)	43.45	348.91
Minerals	58.15	58.15
Vitamin mixture, choline	15.5	15.5
Edible blue dye	0.065	0.065
Total	1 000	1 000
Energy ratio/(kcal %)		
Protein	18.0	20
Fat	10.2	60
Carbohydrates	71.8	20
Total	100	100

**Table 2:** Effects of *Clostridium butyricum* WL-53 on serum and liver lipid metabolism-related indicators in mice (*n* = 5).

Sample Source	Items	Groups			*p*-Value
		ND	HFD	HFD-CB	
Serum	BG mmol/L	1.88 ± 0.30 ^b^	4.01 ± 0.39 ^a^	2.44 ± 0.38 ^b^	0.021
	TC mmol/L	1.51 ± 0.22 ^b^	2.40 ± 0.17 ^a^	1.91 ± 0.19 ^b^	0.036
	TG mmol/L	0.40 ± 0.02 ^b^	0.49 ± 0.02 ^a^	0.44 ± 0.02 ^b^	0.029
	LDL mmol/L	0.19 ± 0.04 ^b^	0.45 ± 0.08 ^a^	0.38 ± 0.07 ^ab^	0.041
	HDL mmol/L	0.96 ± 0.27	1.38 ± 0.31	1.36 ± 0.29	0.521
Liver	TC μmol/g	22.93 ± 6.38 ^b^	55.28 ± 9.82 ^a^	32.78 ± 7.42 ^b^	0.043
	TG μmol/g	176.00 ± 41.20 ^b^	372.47 ± 60.87 ^a^	196.59 ± 46.19 ^b^	0.032
	FFA μmol/g	31.71 ± 3.81 ^b^	48.76 ± 4.86 ^a^	34.80 ± 3.72 ^b^	0.036

Note: Results are expressed as mean ± SEM; SEM = Standard Error of the Mean; Different letters in the same row indicate significant differences (*p* < 0.05). Abbreviations: ND, standard diet group; HFD, high-fat diet group; HFD-CB, high-fat diet treated with *Clostridium butyricum* WL-53 group; BG, blood glucose; TG, triglycerides; TC, total cholesterol; LDL, low-density lipoprotein; HDL, high-density lipoprotein; FFA, free fatty acids.

**Table 3:** The effect of *Clostridium butyricum* WL-53 on serum and colonic cytokines in mice (*n* = 5).

Sample Source	Items	Groups			*p*-Value
		ND	HFD	HFD-CB	
serum	TNF-α (pg/mL)	312.83 ± 14.03 ^b^	372.50 ± 16.12 ^a^	344.59 ± 13.87 ^b^	0.041
	IL-1β (pg/mL)	12.66 ± 0.44 ^b^	14.54 ± 0.51 ^a^	12.94 ± 0.47 ^b^	0.032
	IL-10 (pg/mL)	1098.29 ± 38.90 ^ab^	1000.05 ± 44.58 ^b^	1182.71 ± 39.12 ^a^	0.028
	IL-4 (pg/mL)	123.49 ± 5.24 ^a^	114.69 ± 4.83 ^b^	139.89 ± 5.62 ^a^	0.013
colon	TNF-α (ng/L)	482.85 ± 19.62 ^b^	573.35 ± 24.31 ^a^	501.87 ± 20.07 ^b^	0.024
	IL-10 (ng/L)	133.12 ± 8.61 ^a^	74.09 ± 9.62 ^b^	112.98 ± 8.67 ^ab^	0.017
	IL-22 (ng/L)	25.99 ± 0.94 ^a^	22.16 ± 0.85 ^b^	24.82 ± 0.96 ^a^	0.033

Note: Results are expressed as mean ± SEM; SEM = Standard Error of the Mean; Different letters in the same row indicate significant differences (*p* < 0.05). Abbreviations: ND, standard diet group; HFD, high-fat diet group; HFD-CB, high-fat diet treated with *Clostridium butyricum* WL-53 group; TNF = tumor necrosis factor; IL = interleukin.

## Data Availability

The raw sequencing data generated in this study have been deposited in the NCBI database under the accession number PRJNA1304187.
